# Three-dimensional facial swelling evaluation of piezo-electric vs conventional drilling bur surgery of impacted lower third molar: a randomized clinical trial

**DOI:** 10.1186/s12903-023-02910-6

**Published:** 2023-04-21

**Authors:** A. Caputo, E. Rubino, A. Marcianò, M. Peditto, A. M. Bellocchio, R. Nucera, G. Oteri

**Affiliations:** Department of Biomedical and Dental Sciences and Morphological and Functional Imaging, University of Messina, Via C. Valeria, 98125 Messina, Italy

**Keywords:** Piezosurgery, Conventional osteotomy, Mandibular third molar, Facial scan, 3D digital analysis, Facial diagnostics, Smartphone-based scan, Facial swelling, Bellus 3D Dental pro

## Abstract

**Background:**

Among the post-surgical complications of lower wisdom teeth surgery, swelling is considered by patients one of the most impairing, with both social and biological influences and impacting patients' quality of life. Aim of the study was to evaluate the swelling following the osteotomy when performed with drilling burs versus piezo-electric instruments in the mandibular impacted third molar extraction, using a facial reconstruction software.

**Materials and methods:**

A randomized, split-mouth, single-blind study was conducted on patients, ranging between 18 and 40 years of age, requiring lower third molars extraction and referred at the Oral Surgery Unit of the School of Dentistry of the University of Messina. Twenty-two patients were recruited during an 8 months period according to the following criteria: good general health conditions; bilateral, symmetrical, impacted third molars; no use of medication that would influence or alter wound healing; no temporomandibular joint disorder history; no smoking. All patients underwent bilateral surgical removal. For each patient, a facial scan was obtained prior to the surgical procedures. The two extractions were conducted performing, in a randomized way, osteotomy with rotatory burs or use of piezo surgical instruments. Facial scans were repeated at 3 and 7 days after the surgical procedures. Volumetric differences were calculated via superimposition using a dedicated software. The data obtained were processed using paired t-test.

**Results:**

The results obtained from our study showed no significant differences between two groups regarding post-operative swelling. To the best of our knowledge, this study represents the first experience of using an objective method that can be reproducible on the collection of patients' clinical parameters.

**Conclusions:**

The 3D digital analysis, in the evaluation of facial swelling, is a technique of simple application, objective, reproducible, reliable, decreasing the variables of error.

Based on these data, it is possible to conclude that piezo surgery is a safe way for performing the osteotomies during third molar surgery. However, regarding the post-operative swelling, it does not show an advantage over classical rotary instruments.

**Trial registration:**

Registered on ClinicalTrials.gov (ID: NCT05488028, on 04/08/2022).

Approved by Ethical Committee of Messina: (ID 01–2020, on 27/04/2020).

## Introduction

Included wisdom teeth surgery is one of the most common procedures performed by oral surgeons, usually associated with intraoperative and postoperative complications [1].

The most significant post-surgical complications are pain, swelling, lockjaw, and even paresthesia of the lower lip or tongue, which can have both social and biological impact and can compromise patients' quality of life [2–4]

Conventional surgery using rotary instruments is the most common technique in extraction procedures.

The conventional technique has disadvantages, such as the excessively high temperature produced during osteotomy that can cause marginal bone necrosis and compromise hard and soft tissue healing [5].

In the last decades, technological innovations have been introduced in oral surgery to allow less invasive approaches, ranging from use of piezoelectric instruments to dynamic navigated surgery [6].

In particular, the advent of ultrasound, in surgery, has improved several oral surgical procedures, such as the extraction of impacted third molars.

According to a systematic review with meta-analysis by AL-Moraissi et all. in 2016, the piezo electric surgical technique used in third molar extractions shows a significant reduction in post-operative sequelae (oedema, pain, trismus). The low incidence of post-operative sequelae seems to be related to the atraumatic and micrometric cutting action of the instrument [7–9].

Piezo surgery is effective in osteotomy because it works selectively, being inert against soft tissue, nerves and blood vessels. This represents a significant advantage over a bur [10].

When used appropriately, piezo surgery causes less structural and cellular damage than conventional surgery. In addition, the formation of new bone is faster than with rotary burs [11].

Several studies have shown that the micrometric cutting action of piezo surgery requires a longer intervention time than the use of a bur, potentially causing more discomfort in the postoperative period [12–14].

The aim of this study is to evaluate, in an innovative way, the facial swelling following the osteotomy performed with rotary instrument (R group) versus piezo electric instrument (P group) in the mandibular impacted third molar extraction, using a facial reconstruction software.

## Materials and methods

### Sample and study design

A randomized, split-mouth, single-blind study was conducted on patients referred at the Oral Surgery Unit of the School of Dentistry of the University of Messina, ranging between 18 and 40 years of age and requiring lower third molars extraction. Study protocol was based on an already validated operative scheme [15] and designed according to the CONSORT statement.

Sample size was calculated using the data derived from a preliminary analysis on 10 subjects previously conducted by the authors in order to estimate the considered main outcome (swelling) variation. Values obtained from the preliminary analysis and used to perform the sample size calculation of R group and P group were 1.62 and 1.38 respectively, with a shared standard deviation (σ) of 0.28; power analysis was performed setting α = 0.5 and 0.8 power level. A sample size of 22 subjects was therefore obtained.

Twenty-two patients were recruited during an 8 months period according to the following criteria: good general health conditions; no clinical evidence of major facial asymmetry; presence of bilateral and symmetrical impacted third molars (according to the classifications of Winter and Pell and Gregory); no use of medication that would influence or alter wound healing; no temporomandibular joint disorder history; no smoking.

The patients were included in the study after the registration of personal and clinical data and the collection of TC scan of the teeth to be extracted.

The local Ethical Committee of Messina approved the study protocol (ID 01–2020, on 04/27/2020), in accordance with the Helsinki declarations. The study was registered on ClinicalTrials.gov (ID: NCT05488028).

Patients had given their consent to treatment and were informed that their data would be used for statistical analyses related to this study; informed consent was obtained from all participants.

Randomization was conducted with a table of casual numbers by an investigator who was not part of the study and who was blind to the identity of the procedures.

All patients were enrolled in two groups, P group included all the surgeries carried out with piezoelectric technique, while operations carried out with the bur were assigned to R group.

All patients underwent a 3-d facial scan before the surgical teeth removal using Bellus 3D Dental Pro (Bellus 3D, Inc. 1901 S. Bascom Ave. Suite 1300 Campbell, CA 95,008 USA).

Bellus 3D Dental Pro is a dental app for iOS devices that uses the integrated to scan and reproduce the face of a subject with a 3-dimensional render in less than 15 s. The facial scan can be subsequently exported in various formats, such as STL.

For each patient, the impacted teeth were extracted in two different phases, separated by a 30-day time interval. All procedures were performed by a single experienced oral surgeon.

The study involved three time points:

• reference scan (T0), face scan before surgery, at time 0;
• target scans (T1) and (T2), respectively at 3 and 7 days, after each surgery, both with rotating and piezoelectric instrumentation for a total of 5 scans.

The Esacrom Piezosurgery device (Esacrom electronics and medical devices, Imola, Italy) was used for ultrasonic osteotomies according to the manufacturer's instructions using a specific insert for osteotomies (ES07WT).

The Lindemann stainless steel bur (shank diameter 2.35 mm; length 44 mm) mounted on a high-speed straight surgical handpiece was used for osteotomies with conventional technique.

### Study outcome measures

Main outcome of the study was the evaluation of postoperative facial swelling (assessed via digital comparison of facial scans obtained at 3 and 7 days after lower third molar removal to a presurgical baseline scan) using different surgical techniques.

### Surgical and post-surgical variables

**Fig. 1:**
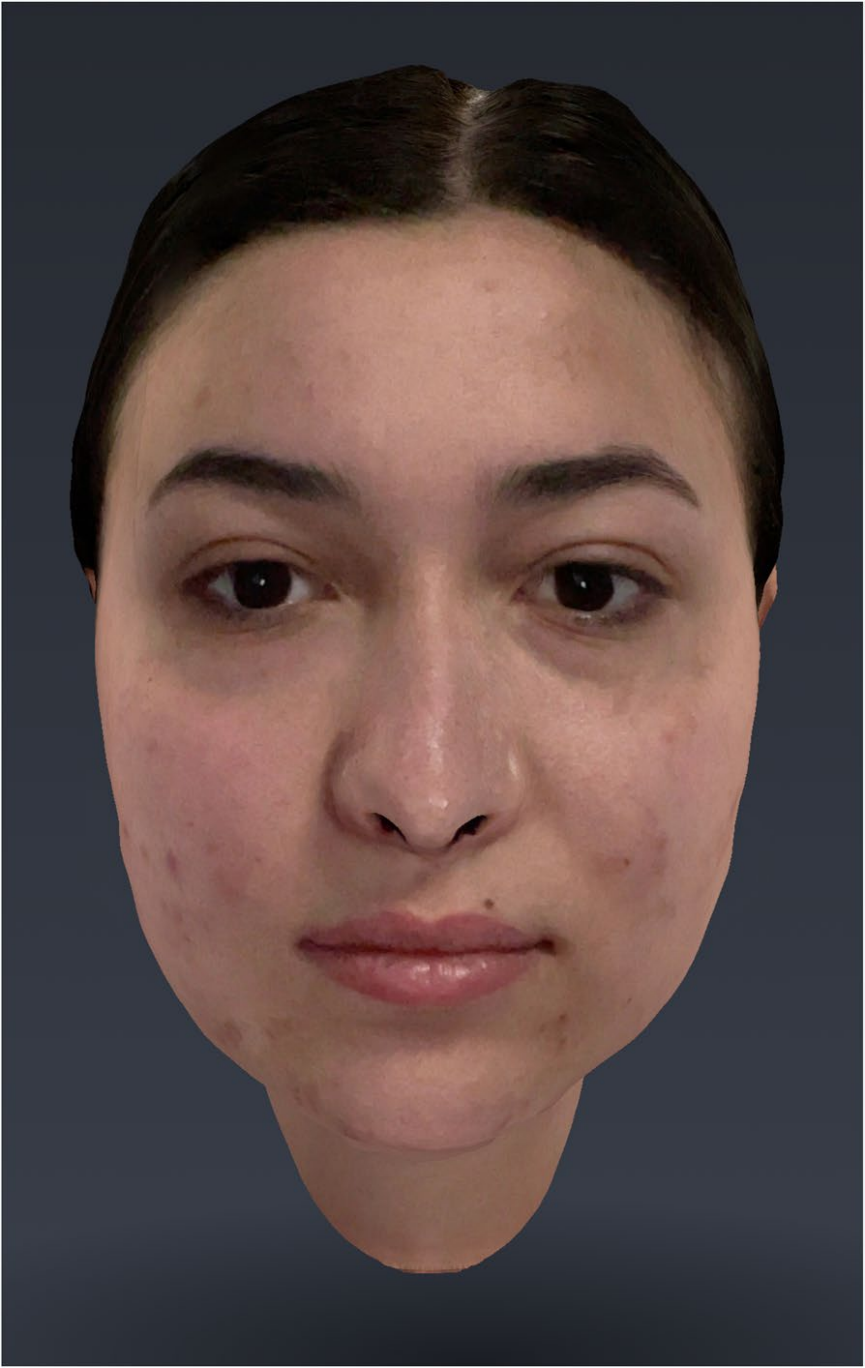
Scan T0

Preoperatively, all patients underwent three-dimensional facial scanning (Fig. 1).

Three-dimensional images were captured by the Bellus 3D Dental Pro app.

The first extraction of the included lower third molar was performed.

**Fig. 2:**
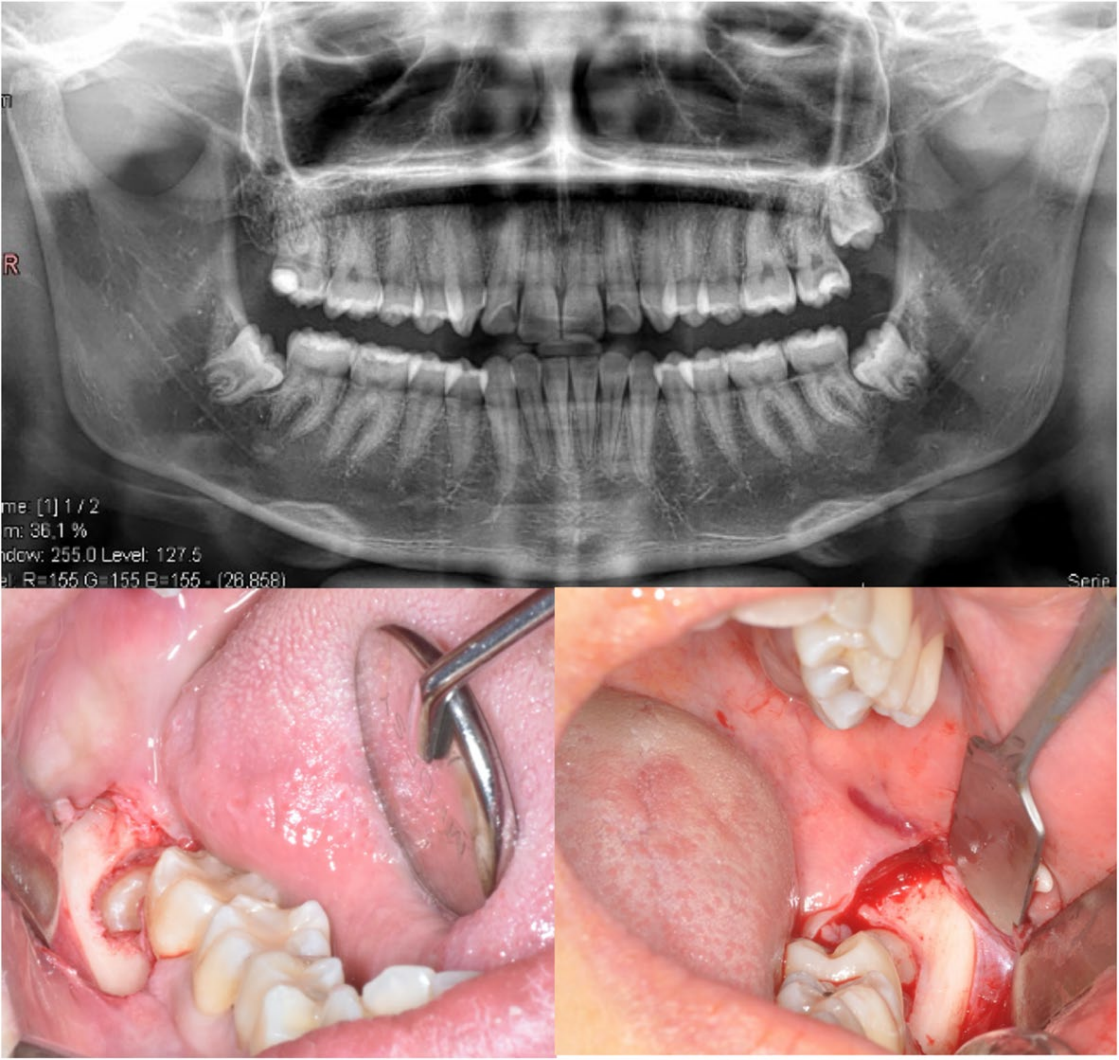
Sample of the surgery of symmetrical inferior impacted third molars

Each of the two extractions was conducted using standardized procedures. Nerve block of the inferior alveolar and buccal nerve with mepivacaine hydrochloride 3% with adrenaline 1:100,000 was performed. A full-thickness envelope flap with a vertical releasing incision was reflected, and osteotomy were subsequently performed (Fig. 2).

Both the side of the surgery and the technique to be used were decided at random. The osteotomy was performed on one side with piezoelectric instrumentation and the other side with rotary instrumentation.

When necessary, tooth sectioning was performed with a high-speed tungsten carbide slit drill under saline irrigation and the tooth removed in single or multiple segments.

The mucoperiosteal flap was repositioned and the surgical wound was closed with a 5–0.

After surgery, the pharmacological therapy was prescribed to each patient for each intervention (Amoxicillin 1gr cpr with posology 1 cpr every 12 h for 6 days; in case of allergy to penicillins, clarithromycin 500 mg with posology 1 cpr every 12 h for 6 days was prescribed; chlorhexidine mouthwash at 0.20% to be used three times a day for 10 days after surgery, to reduce the bacterial load). The patient received all indications regarding postoperative management.

**Fig. 3:**
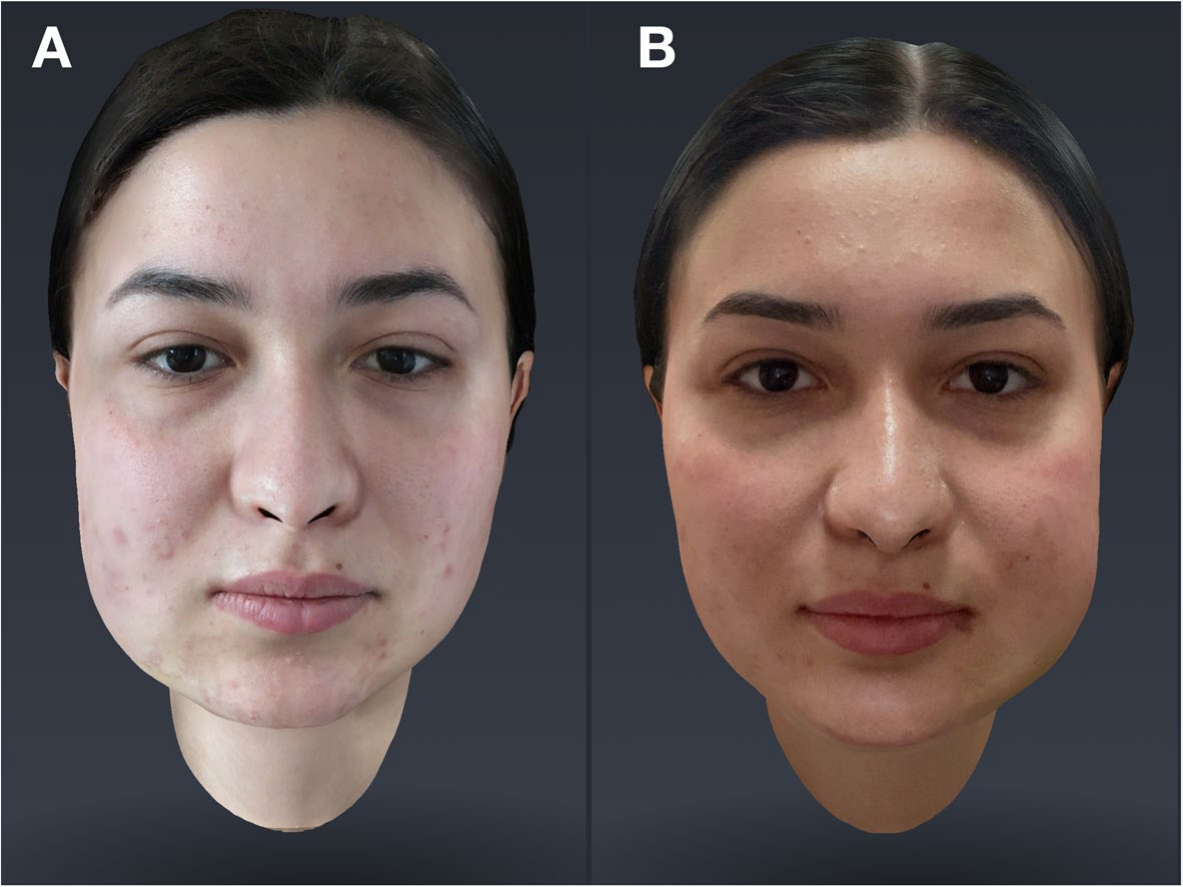
Scan T1(P-R)

Three days after each of the two surgeries, the second 3D scan of the face (T1) was performed to assess facial swelling (Fig. 3).

**Fig. 4:**
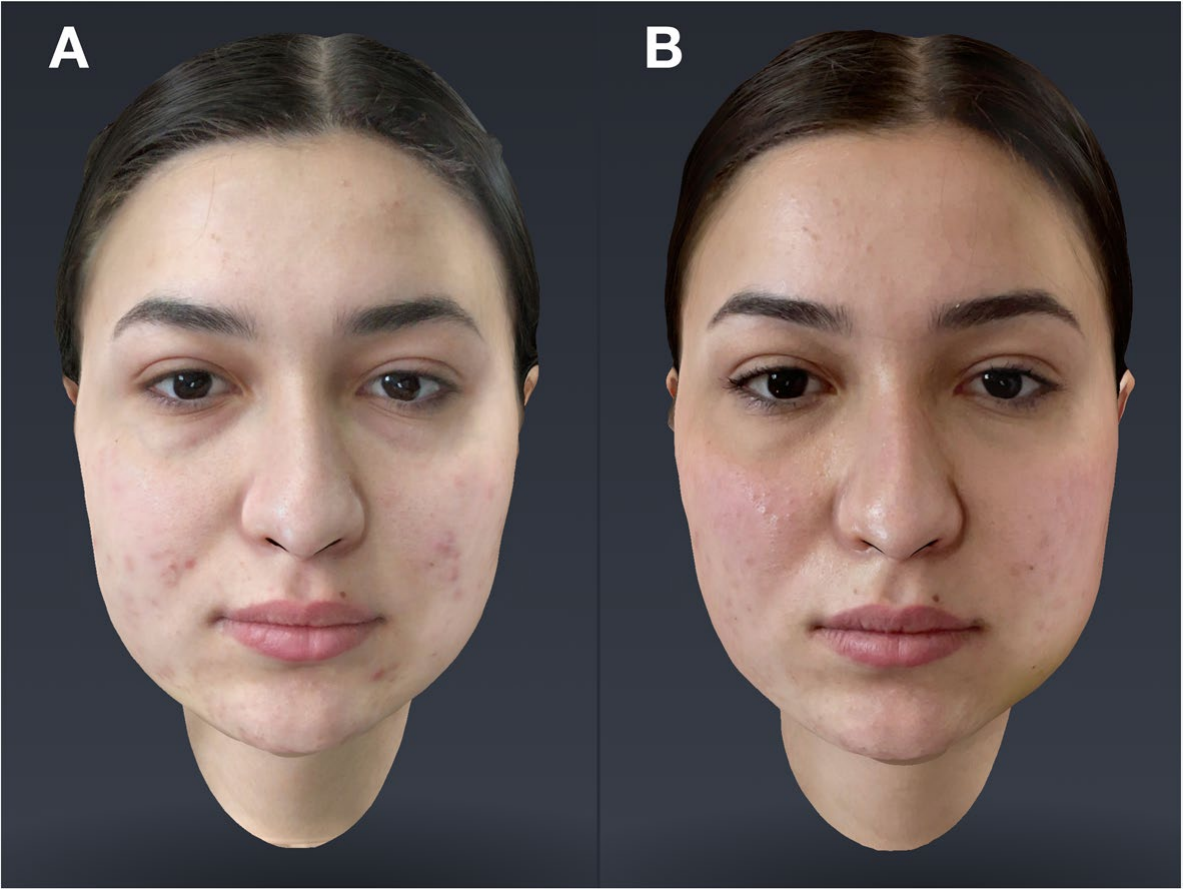
Scan T2(P-R)

Scans (T2) were performed at 7 days after each surgery (Fig. 4).

### 3d images evaluation

Scans were exported in STL (Standard Triangulation Language) files and imported within a dental application software “Medit Compare” (MEDIT corp. 23 Goryeodae-ro 22 gil, Seongbuk-gu, Seoul, Korea).

Medit Model Builder software allows the user to create physical models from digital facial scans.

**Fig. 5:**
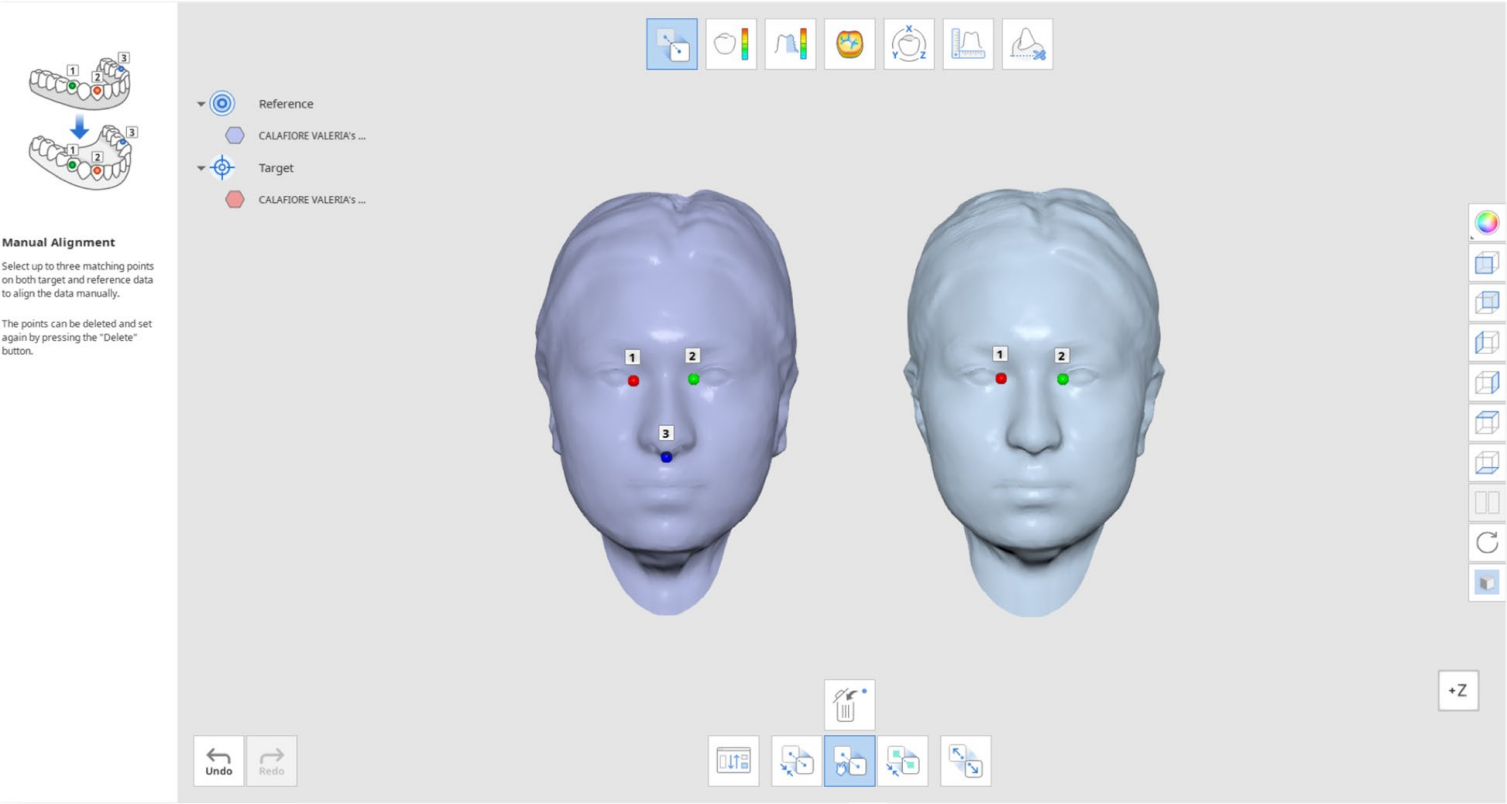
T0-T1 and T0-T2 scans superimposition

T0-T1 and T0-T2 scans were opened and superimposed through three reference points:

Endocanthion left (inner most point on commissure of left eye fissure), endocanthion right (inner most point on commissure of right eye fissure) and subnasale (mid-point of columella) (Fig. 5).

### Data analysis

Scans were exported in STL "Standard Triangulation Language" files and imported within a dental application software. Statistical analyses were carried out using Minitab, version 21.1.

Data are summarized as mean ± standard deviation.

A t Test was conducted twice to compare the averages and see if there is a significant difference between the averages of the groups. *P* < 0.05 was considered to indicate a statistically significant difference.

## Results

A total of 22 patients (16 F, 6 M), aged 18 to 40 years, with symmetrical impacted lower third molars were included in the study. Their mean age was 24.70 years.

No cases of post extraction alveolitis or site infection were reported during follow-up, and no adverse drug reactions were observed.

Data regarding facial swelling measured comparing T1 and T2 facial scan to baseline (T0) are reported in Table 1 as mean values.

**Table 1:** Average measurements of the 22 patients at T0-T1 and T0-T2 (R) and (P)

	AVRG	AVRG	AVRG	AVRG
PATIENTS	T0-T1 (R)	T0-T2 (R)	T0-T1 (P)	T0-T2 (P)
1	1,414	1,002	0,002	0,772
2	0,864	0,599	0,865	0,477
3	1,834	1,597	1,942	1,043
4	1,652	1,301	1,266	0,952
5	0,84	0,585	1,537	0,661
6	1,657	0,935	1,100	0,824
7	1,892	1,757	1,856	1,541
8	1,447	0,728	0,768	0,59
9	1,246	1,009	0,966	0,762
10	1,522	0,909	1,648	1,027
11	1,238	1,082	1,605	1,256
12	0,802	0,366	1,014	0,72
13	1,243	1,105	0,966	0,667
14	2,022	0,74	1,563	0,749
15	2,866	1,065	2,298	1,458
16	1,786	1,302	1,601	0,487
17	2,516	1,342	1,517	1,361
18	2,617	0,928	1,676	1,263
19	1,898	1,702	1,852	1,498
20	1,642	1,288	1,302	0,841
21	0,901	0,456	1,021	0,562
22	1,422	0,802	1,348	0,781

**Fig. 6:**
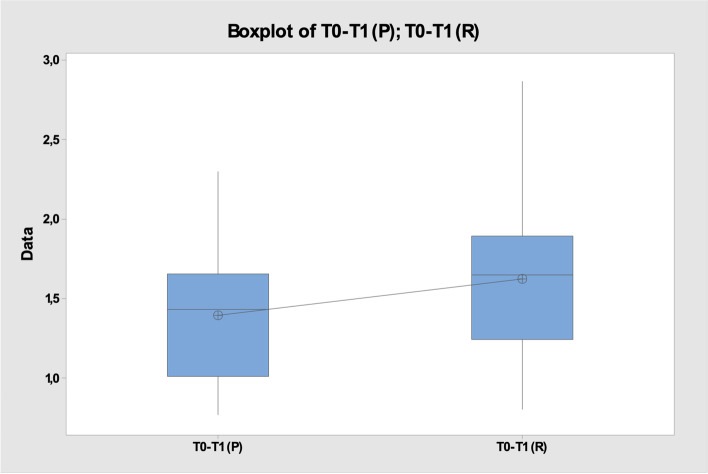
Mean ± standard deviation T0-T1(P) vs T0-T1(R)

**Fig. 7:**
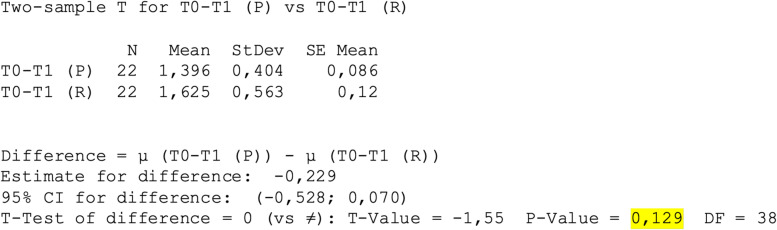
Two-Sample T-Test and CI: T0-T1 (P); T0-T1 (R)

Among the considered sample, the average of the mean values obtained from matching the T0-T1(R) scans for the rotating instruments was 1.66 ± 0.6, whereas in the case of the piezosurgery the average of T0-T1(P) was 1.39 ± 0.4 (Fig. 6), *p* > 0.05 (Fig. 7).

**Fig. 8:**
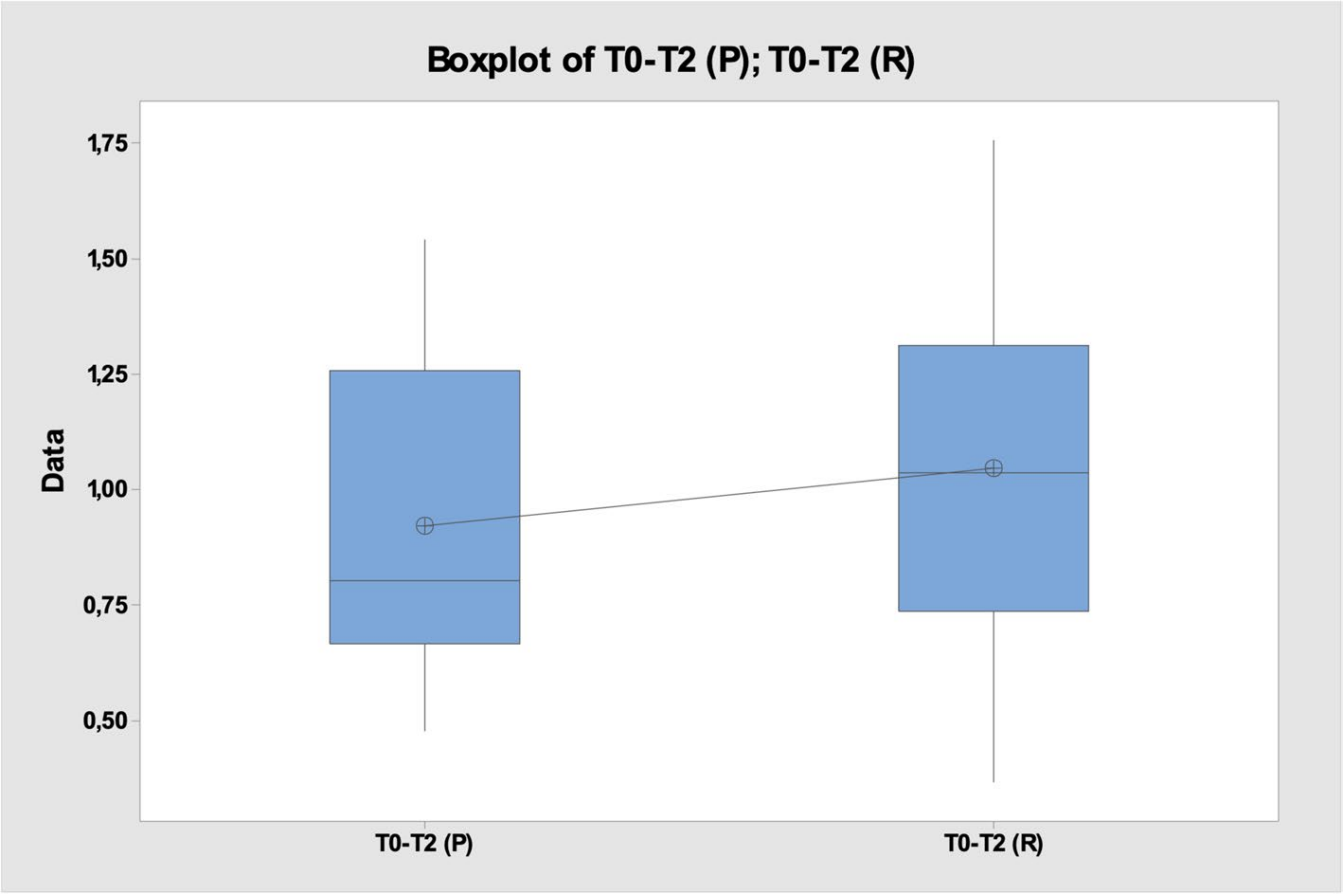
Mean ± standard deviation T0-T2(P) vs T0-T2(R)

**Fig. 9:**
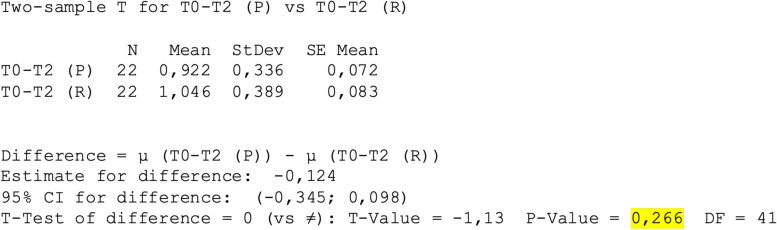
Two-Sample T-Test and CI: T0-T2 (P); T0-T2 (R)

Regarding the assessment of swelling at T0-T2, the mean of the recorded values was T0-T2(P) 0.92 ± 0.3 and T0-T2(R) 1.04 ± 0.4 (Fig. 8), *p* > 0.05 (Fig. 9).

The two-sample t Test showed no statistically significant variation between groups at any given timepoint in regards to postoperative swelling.

## Discussion

Third molar surgery can be complicated. Clinicians' priority is to promote optimal treatment outcomes while maintaining the integrity of the noble structures.

In oral surgery, conventional instrumentation for bone removal is performed with rotary handpieces. In recent years, piezo surgery has gained popularity. It has been considered safe and effective and uses micro vibrations with ultrasonic frequency [10]. It has a 'selective cutting' because it only works on mineralized structures, and this allows a safe osteotomy to be performed while protecting the noble structures [16]

It promotes bone healing as it does not produce high temperatures [17] and it has a constant irrigation system [18].

However, the operating time is longer compared to the use of rotary instruments [19]. This led researchers to compare the different effects of piezoelectric and conventional instrumentation on postoperative morbidity in third molar surgery. In a randomized study, it was shown that pain, swelling and lockjaw were reduced in patients treated with piezoelectric instrumentation, while the duration of surgery was longer [20]. Third molar extraction, even when planned and performed by an experienced operator, is not without its complications; in fact, it is frequently associated with significant post-surgical sequelae (4.6% to 30.9%), particularly pain and swelling, which can have both social and biological impact[2, 3, 21]. Considering their impact on patient’s quality of life, different Authors have investigated adjuvant systems, such as probiotics, to improve post-surgical outcomes and healing processes [22, 23].

It is necessary to differentiate true complications from sequelae that are part of the postoperative course such as pain, swelling, trismus, moderate bleeding, and hematoma. These complaints are typically present in 35% of cases during the first postoperative day, 25% at 7 days, and 4% at 14 days [24].

The incidence of complications depends on the difficulty of treatment, the severity of inclusion, and the age of the patient [25, 26].

Some authors have shown that pain and swelling are directly proportional to the difficulty of the procedure and the treatment time [3, 27, 28].

In this split-mouth study, postoperative swelling was evaluated with facial 3d scan by comparing two different surgical techniques (piezo surgery vs. rotary instruments) in the extraction of impacted third molars.

According to Winter and Pell and Gregory's classifications, included third molars were evaluated.

Only patients with the same classification for third molars were enrolled.

A homogeneous sample of patients and teeth was selected as age, position and anatomy may influence the postoperative course.

Post-surgical facial swelling is difficult to accurately quantify because measurements are made on an irregular surface.

Several papers have assessed postoperative swelling, after lower third molar surgery, by face measurements with manual techniques, obtained with a tape measure [29–31]

However, this technique is operator-dependent and for that reason not objective.

To date, there is no accurate and reliable measurement method in the literature for evaluating facial swelling after third molar surgery.

In this study, innovative, digital measurements were used that allowed us to obtain reliable data for objective comparison.

The Bellus 3D application, for scanning patients' faces, and the dental application software for obtaining the results, showed ease of use.

The results obtained from our study showed no significant differences between two groups regarding postoperative swelling which contradict with other reports in the literature [20, 32].

We argue that this may be related to the reduced number of patients.

To the best of our knowledge, this study represents the first experience of using an objective method that can be reproducible on the collection of patients' clinical parameters, on the other hand, the use of the application and software has financial implications.

Consequently, this study is added to others in a constant effort to obtain increasingly reliable data. The main limitation of our study is represented by the small sample size. However, while image acquisition through the Bellus 3D app can be considered a very simple and straightforward procedure, image processing to evaluate swelling requires a well-versed operator and may present a steep learning curve. Moreover, every surgical procedure was performed by a single experienced oral surgeon, so results may vary together with operator skills.

## Conclusion

This study showed a new method to record clinical data of patients after surgery of impacted lower third molars, a frequent condition observed in the general adult population.

The 3D digital analysis, in the evaluation of facial swelling, is a technique of simple application, objective, reproducible, reliable, decreasing the variables of error.

It represents a valid alternative to the manual techniques used until now, thus reducing operating times.

Based on these data, it is possible to conclude that piezo surgery is a safe way for performing the osteotomies during third molar surgery. However, regarding the postoperative swelling, it does not have an advantage over classical rotary instruments.

## Data Availability

Study dataset can be find at the following link: https://drive.google.com/drive/u/1/folders/1OfErb2lFRrSkGYhB8qb07THEtH1xWGF1 Missing data from 4 patients were not uploaded due to technical problems (files were either corrupted or unretrievable from our archives).
